# Effects of Mental Imagery on Quality of Life, Cognitive, and Emotional Status in Older Adults with Early-Stage Alzheimer’s Disease Dementia: A Randomized Controlled Trial †

**DOI:** 10.3390/brainsci14121260

**Published:** 2024-12-16

**Authors:** Anna Christakou, Christina Bouzineki, Marousa Pavlou, George Stranjalis, Vasiliki Sakellari

**Affiliations:** 1 Department of Physiotherapy, School of Health Sciences, University of West Attica, 12243 Athens, Greece; achristakou@uniwa.gr; 2 Laboratory of Biomechanics, Department of Physiotherapy, School of Health Sciences, University of Peloponnese, 23100 Sparta, Greece; 3 Marousi Day Center Alzheimer Athens Association, 11636 Athens, Greece; cbouzineki@yahoo.com; 4 Centre for Human & Applied Physiological Sciences, King’s College, London WC2R 2LS, UK; marousa.pavlou@gmail.com; 5 Department of Neurosurgery, University of Athens, Evangelismos Hospital, 10676 Athens, Greece; stranjal@otenet.gr

**Keywords:** dementia, imagery, physiotherapy, quality of life, cognitive status, emotional status

## Abstract

Background/Objectives: Dementia is a syndrome which involves cognitive and motor problems such as memory and motor control that impacts the individuals’ quality of life. In mental imagery (MI) technique, motor acts are mentally rehearsed without any overt body movements. The aim of this study was to investigate the effectiveness of MI on the quality of life, cognitive, and emotional status of older adults with an early stage of dementia. Methods: The present randomized controlled trial consisted of 160 participants. The sample from an Athens Day Care Center of the Alzheimer Association was randomized to (a) the MI and exercise group (intervention group, n = 55), (b) the only exercise group (1st control group, n = 52), and (c) the neither MI nor exercise group (2nd control group, n = 53). Participants engaged in a total of 24 physiotherapy sessions, each lasting approximately 45 min, scheduled twice weekly over a 12-week period. They performed three assessments: (a) one week prior to the program, (b) one and a half months, and (c) after the program. The intervention group performed a 30 min MI with exercise program content immediately following every physiotherapy exercise session. Walking While Talking Test (WWITT), the Euro-Qol 5-Dimensions 5-Level of severity scale (Euro-Qol 5D-5L scale), the Short-Form of Geriatric Depression Scale (SF-GDS), and the Short Anxiety Screening Test (SAST) were used to assess cognitive status, emotional status, and quality of life. Results: A total of 160 participants (43 men, 117 women, with MMSE M = 23.20 SD = 0.15) took part in this study in which most reported holding a university degree (n = 77), were married (n = 101), and had loss of memory as the 1st symptom of dementia (n= 117). The Friedman test revealed statistically significant differences across the three groups on (a) the WWITT_mistakes_ (X^2^ = 14.95, df = 2, *p* = 0.001) and WWITT_time_ (X^2^ = 13.35, df = 2, *p* = 0.01), (b) the total Euro-Qol 5D-5L scale (X^2^ = 11.87.62, df = 2, *p* = 0.003) and quality of life on the measuring day (X^2^ = 25.59, df = 2, *p* = 0.00), (c) the SF-GDS (X^2^ = 6.54, df = 2, *p* = 0.038), and (d) the SAST (X^2^ = 39.907.62, df = 2, *p* = 0.00). The Friedman test with post hoc Wilcoxon analysis revealed that the mean scores for the intervention group and the 1st control were significantly better than the 2nd control group in many dependent variables. Conclusions: The results indicate that incorporating MI can positively influence cognitive status, emotional status, and the quality of life in older adults diagnosed with early-stage dementia.

## 1. Introduction

The progression from normal cognition to Alzheimer’s disease dementia (ADd) has become a significant public health concern, drawing increasing attention from healthcare providers, clinicians, and researchers. This transition often includes an intermediate stage, known as amnestic mild cognitive impairment (aMCI), which is widely regarded as a precursor to ADd. Dementia is marked by a reduction in cognitive functions, particularly memory impairments, which can adversely affect overall well-being, mood, and self-image, ultimately diminishing quality of life in daily activities [1].

Exercise, as a non-drug therapeutic approach, prevents cognitive decline and enhances the quality of life for individuals with cognitive impairment or dementia [2]. Many randomized controlled trials have highlighted the beneficial impact of exercise on cognitive function, activities of daily living (ADLs), and neuropsychiatric symptoms within this population [3,4]. Research indicates that aerobic and resistance training may influence cognitive health through distinct molecular mechanisms. Therefore, the choice of exercise type is a critical consideration for clinicians when designing interventions to prevent or mitigate cognitive decline in dementia [5].

However, the optimal type of exercise for preventing or slowing cognitive decline remains uncertain, as direct comparisons of different exercise interventions are lacking. This uncertainty poses challenges for healthcare professionals in prescribing the most effective exercise regimens for this population. Given the inconsistent findings regarding exercise types, recent research has turned to complementary and alternative medicine (CAM). CAM encompasses traditional, holistic, and alternative practices, which may be used alongside or in place of conventional pharmacological treatments and physiotherapy. Among these, ‘mind-body medicine’ therapies within CAM have garnered growing interest [6].

Mental imagery (MI) is a mind-body technique where individuals visualize themselves executing an action, preferably from a first-person viewpoint, without engaging in any actual muscular movement, and then proceed to execute the action [7,8]. This approach facilitates motor learning by stimulating the Mirror Neuron System. Research indicates that when the imagined and executed actions have similar durations, neuroplasticity is enhanced [9]. During MI, brain activation engages a distributed premotor-parietal network and several subcortical structures, including the putamen and cerebellum [10,11].

MI contributes in neurological rehabilitation, particularly in stroke [12], Parkinson’s disease [13], and multiple sclerosis [14]. It has been shown to enhance gait speed, gait performance, balance, cognitive abilities, daily living activities, and overall quality of life. MI presents several advantages: it is non-invasive, safe, cost-effective, and can be conveniently performed in house [15].

To date, no studies have investigated the impact of MI on the emotional status, cognitive function, and quality of life of older adults with early-stage dementia. Experimental research investigating the psychophysiological processes underlying MI in the context of dementia remains scarce. Understanding these processes is crucial not only for theoretical advancements but also for their clinical implications. Such insights could help physiotherapists integrate MI into rehabilitation programs, thereby contributing to participants’ cognitive function and overall well-being. Accordingly, the present study aims to evaluate the effects of MI on the emotional status, cognitive function, and quality of life of older adults with early-stage dementia.

## 2. Materials and Methods

### 2.1. Study Design

A single-blind randomized controlled trial (RCT) was conducted in accordance with the Declaration of Helsinki and Good Clinical Practice (GCP). Both the Ethics Committee of the University of West Attica (study protocol: 93292—26 October 2021) and the Day Center Alzheimer Athens Association, Athens, Greece (protocol number: 150—7 July 2021) approved the initiation of this study. The trial was registered with ClinicalTrials.gov (accessed on 13 January 2022) under the identifier NCT05232526.

### 2.2. Sample

Using G*Power version 3, the sample size was calculated with an effect size of 0.9, a power of 0.8, and an alpha (α) error of 0.05. An a priori power analysis was conducted for a one-tailed t-test comparing independent means. A non-probability convenience sampling method was employed, resulting in the recruitment of 160 participants from the Day Care Centers of the Athens Alzheimer Association between September 2021 and June 2024. Of these, 142 participants completed the intervention. This study included older adults aged 65 to 95 years with early-stage dementia. Participants were randomly assigned to one of three groups using a lot-drawing method: the experimental group (EG), which participated in both a mental imagery (MI) and exercise program; the first control group (CG1), which participated only in the exercise program; and the second control group (CG2), which received neither MI nor an exercise program. One researcher oversaw participant enrollment and group allocation. The EG took part in a 4-week intervention program alongside their regular physiotherapy exercise routine. During the baseline assessment, the demographic data were collected. Inclusion criteria included the following: (a) Age between 65 and 95 years; (b) Diagnosis of early-stage Alzheimer’s-type dementia by a neurologist at the Athens Alzheimer Association Day Care Center, based on the International Statistical Classification of Diseases and Related Health Problems (ICD-10) Version 2019 codes F00, F01–F03, and a Mini-Mental State Examination (MMSE) score ranging from 20 to 25; (c) Additionally, candidates were required to demonstrate oral and written communication abilities to follow instructions effectively; (d) Ambulatory status; and (e) No significant health issues in the preceding month. Both sexes were included. Exclusion criteria comprised the following: (a) Late-stage dementia; (b) Psychiatric disorders; (c) Cardiovascular or pulmonary conditions; (d) Concurrent presence of other neurological disorders, including Lewy body or vascular dementia; and (e) Inability to walk. Participants completed an information sheet and signed a consent form before enrollment.

### 2.3. Outcomes

Outcome measures were assessed at three distinct time points over a period of roughly three months: (1) an initial baseline assessment conducted before to the intervention period (pre-intervention), (2) a mid-point assessment at the conclusion of the 8th week of the intervention program, and (3) a post-assessment conducted at the conclusion of the 12th week of the intervention. The measures aimed to assess changes in various domains, including cognitive ability, as outlined below.

#### 2.3.1. Cognitive Ability

The Walking While Talking Test (WWTT) was utilized as a dual-task assessment to measure cognitive–motor performance. During the WWTT, participants walked at a self-paced speed over a 6 m distance, turned, and walked back to the starting point. Concurrently, they recited 12 Greek alphabet letters, starting with “A” (A, Β, Γ, Δ, Κ, Λ, Μ, Ν, Π, Ρ, Σ, Τ), aligning each letter to a step. The test assessed the duration required to finish the task and also noted any errors made while reciting the alphabet [16].

#### 2.3.2. Depression

The Short-Form Geriatric Depression Scale (SF-GDS) is a 15-item assessment tool developed to evaluate depression in older adults. It features a straightforward “Yes/No” response format, making it both user-friendly and efficient for implementation in clinical and research contexts. The tool takes around 5 to 7 min to complete and can be administered by researchers with minimal training [17].

#### 2.3.3. Anxiety

The Short Anxiety Screening Test (SAST) assesses somatic symptoms often associated with anxiety in older adults. The scale is composed of 10 items, each assessed on a 4-point scale, with response choices ranging from ‘rarely or never’ to ‘always’. Total scores range from 10 to 40, with higher scores indicating greater levels of anxiety. A score of ≥24 serves as the cut-off for an anxiety diagnosis, while scores between 22 and 23 are considered borderline. The SAST takes approximately 10 to 15 min to complete, and the total score is calculated by summing the item ratings. The scale demonstrates good psychometric properties. Internal consistency is high, with a reliability coefficient of 0.763 (95% CI: 0.71–0.82, *p* < 0.001). The test-retest reliability was assessed as ‘very good’, with a value of 0.930 (95% CI: 0.918–0.942, *p* < 0.0001). The reproducibility, as measured by the Intraclass Correlation Coefficient (ICC), was 0.763 (95% CI: 0.686–0.827). The SAST also exhibits strong discriminant and factorial validity in the Greek population [18].

#### 2.3.4. Quality of Life

The Euro-Qol 5 Dimensions 5 Levels (EQ-5D-5L), a preference-based instrument, evaluates Health-Related Quality of Life (HRQoL) across five domains: mobility, self-care, usual activities, pain/discomfort, and anxiety/depression. Participants rate each domain on a 5-point scale, where 1 represents the best possible health state and 5 represents the poorest. Additionally, the EQ-5D-5L includes a Visual Analog Scale (VAS), enabling participants to assess their overall health status on a scale ranging from 0 to 100. A score of (a) 0 shows the worst imaginable health state, and (b) 100 shows the best imaginable health state.

The combination of domain scores and the VAS rating provides a comprehensive assessment of the patient’s perceived quality of life [19].

### 2.4. Intervention Description

This study involved implementing a combined mental imagery and exercise program which included the following:

(a) MI program

MI was introduced concurrently with the commencement of the exercise program. The experimental group engaged in 24 sessions, each consisting of 30 min of imagery practice, starting from the first exercise session. The imagery sessions took place in a quiet area following the exercise sessions at the Marousi Day Center, Alzheimer Athens Association. During these sessions, participants were instructed to visualize the exercises they had just completed in the exercise program. All imagery sessions followed a standardized approach for every participant in the experimental group.

(b) Physiotherapy exercise program

The same experienced physiotherapist oversaw the exercise program for both the experimental group and the first control group at the Marousi Day Care Centers of the Athens Alzheimer Association. Over a span of three months (12 weeks), participants engaged in 24 physiotherapy sessions, each lasting 45 min, held twice weekly. The exercise regimen was based on the Otago Exercise Program (OEP) and consisted of the following elements:

(a) Warm-up: Exercises designed to enhance circulation, including joint mobilization and muscle stretching.
(b) Strength and Endurance Exercises: Exercises focused on muscular strength and endurance, performed with or without weights, emphasizing slow and controlled movements within each participant’s range of motion.
(c) Balance Training: Dynamic and static balance exercises aimed at improving posture, supporting daily activities, and building confidence to reduce the risk of falls.
(d) Cool-Down: Stretching exercises aimed at improving flexibility, promoting relaxation, and alleviating fatigue, helping to revitalize the body after each session. The program included exercises like gentle marching, neck movements, back extensions, ankle rotations, front and back knee strengthening, lateral hip strengthening, calf raises, toe raises, heel-to-toe walking, single-leg stances, sideway walking, sit-to-stand transitions, as well as stretches for the back of the thighs and calves [20].

All participants followed the same exercise routine throughout the program, gradually progressing to more advanced exercises as they demonstrated competence at each stage. In the intervention group, participants were assessed for their ability to use mental imagery by completing the Vividness of Movement Imagery Questionnaire (VMIQ). This questionnaire consists of 24 items that evaluate movement imagery, focusing on both visual imagery and kinesthetic sensations. Participants were asked to visualize themselves performing various movements and also to imagine others executing the same motions. The items are categorized into six factors, each containing four items: Basic movements, Basic movements with greater precision, Movements with control but some unplanned risk, Movements involving the manipulation of objects, Movements that induce imbalance and recovery, and Movements requiring control in aerial situations.

The Vividness of Motor Imagery Questionnaire (VMIQ) utilizes a 5-point scale where 1 indicates “completely clear and as vivid as normal vision”, and 5 signifies “no image at all, only a mental acknowledgment of the action”. In the initial four sessions of the intervention phase, the first author explained the concept of imagery to the participants, highlighting its impact on specific populations. During these sessions, participants practiced exercises while receiving guidance to improve their imagery abilities, emphasizing self-awareness, clarity, and control. This process enabled participants to develop the skills to generate, manage, and vividly imagine mental images. Prior to each imagery session, participants engaged in a relaxation exercise, which has been shown to improve the vividness and clarity of imagery. At the conclusion of each session, participants completed a manipulation check using a Likert scale (1 = “not at all,” 5 = “very much”) to confirm they were effectively and accurately visualizing the content.

The evaluations were carried out by a physiotherapist experienced in working with individuals with early-stage dementia. This physiotherapist received training on the study protocols and had a thorough understanding of the study tools. To ensure impartiality, musculoskeletal assessments were performed by an independent assessor who was blinded to the participants’ group allocations.

### 2.5. Statistical Analysis

Data are reported as the mean ± standard deviation for continuous variables, and frequencies and percentages for categorical variables. To assess homogeneity between groups, an independent samples t-test was used for continuous data, while the Chi-square (χ^2^) test was applied for categorical data, with a significance level set at α = 0.05. The normality of the data was examined using the Kolmogorov–Smirnov test. An intention-to-treat approach was implemented to address potential dropouts, utilizing the last observation carried forward (LOCF) method. For non-parametric data, the Friedman test was applied to analyze repeated measures for the following variables:

(a) Walking While Talking (WWT) mistakes;
(b) Walking While Talking (WWT) time;
(c) EuroQoL scores;
(d) EuroQoL scores for the day of measurement;
(e) Depression scores;
(f) Anxiety scores.

The post hoc comparisons were conducted using the Wilcoxon signed-rank test. The effect size for the F-tests was assessed using partial Eta squared (η^2^), where values of 0.01, 0.06, and 0.14 represented small, medium, and large effect sizes, respectively. A two-sided significance level was set at *p* < 0.05 for all tests. The analyses were performed using SPSS version 29 (IBM Corporation, Somers, NY, USA).

## 3. Results

Table 1 displays the demographic and clinical details of the participants. A total of 160 individuals (43 males, 117 females; Mean age = 77.94 years, SD = 7.19; MMSE = 23.20, SD = 0.15) were included in this study. Memory loss was the most frequently reported initial symptom, noted by 137 participants. At baseline, no significant differences were observed across the three groups regarding demographic and clinical characteristics (Table 1).

Eighteen participants were unable to complete this study due to various factors, including fractures (two participants), deaths (seven participants), transportation issues (five participants), COVID-19 (two participants), and weather-related challenges (two participants). As a result, an intention-to-treat analysis was conducted, employing the last observation carried forward (LOCF) method to account for dropouts.

The measurements of the (a) Walking While Talking_mistakes_ and Walking While Talking_time_ (b) the EuroQuol total and EuroQuol on the day, (c) Depression, (d) Anxiety were not normally distributed.

A statistical comparison of the repeated measures was conducted using Friedman’s test, which revealed a significant difference between the three groups in the following:

(a) Walking While Talking _mistakes_ (X^2^ = 14.95 df = 2, *p* = 0.001),
(b) Walking While Talking _time_ (X^2^ = 13.35 df = 2, *p* = 0.01),
(c) EuroQuol total (X^2^ = 11.87.62, df = 2, *p* = 0.003),
(d) EuroQuol on the day of the measurement (X^2^ = 25.59, df = 2, *p* = 0.00),
(e) Depression (X^2^ = 6.54, df = 2, *p* = 0.038),
(f) Anxiety (X^2^ = 39.90, df = 2, *p* = 0.00) (Table 2).

A post hoc analysis using the Wilcoxon signed-rank test with a Bonferroni correction setting significance at *p* < 0.000 revealed the following mean score trends:

(a) Walking While Talking_mistakes:_ Significant differences were observed between the experimental group and the 2nd group (Z = −5.700, *p* = 0.001), and between the experimental group and the 3rd group (Z = −3.359, *p* = 0.001).
(b) Walking While Talking_time:_ Significant differences were observed between the experimental group and the 2nd group (Z = −3.145, *p* = 0.002) and between the experimental group and the 3rd group (Z = −4.096, *p* = 0.001).
(c) EuroQol total index: Significant differences were observed between the experimental group and the 3rd group (Z = −2.354, *p* = 0.02).
(d) EuroQol on the day’s measurement: Significant differences were observed between the experimental group and the 2nd group (Z = −2.097, *p* = 0.036) and between the experimental group and the 3rd group (Z = −4.504, *p* = 0.001).
(e) Depression: Significant differences were observed between the experimental group and the 2nd group (Z = −2.019, *p* = 0.043).
(f) Anxiety index: Significant differences were observed between the experimental group and the 2nd group (Z = −4.089, *p* = 0.001) and between the experimental group and the 3rd group (Z = −3.993, *p* = 0.001).

The scores from the Vividness Movement Imagery Questionnaire, which assesses the ability to create mental images, ranged from 24 (indicating the highest imagery ability) to 120 (indicating the lowest). For the “watching somebody else” condition, scores ranged from 46 to 90 (M = 60.80, SD = 10.72), whereas for the “doing it himself/herself” condition, scores ranged from 38 to 78 (M = 50.00, SD = 10.70). Most participants described the vividness of the imagery in the “doing it himself/herself” condition as clear and realistic. Overall, imagery ability scores varied between 26.2 and 36.4 (M = 30.76, SD = 2.25), with participants generally reporting that their exercise performance felt fairly to very vivid and clear.

## 4. Discussion

The purpose of this study was to assess the effectiveness of MI on cognitive performance, emotional status, and quality of life of older adults with dementia. The results indicated that participants in the experimental group showed improvements in quality of life, along with reductions in depression and anxiety, as well as better cognitive status compared to the two control groups.

MI has been shown to improve emotional stability and memory in individuals with mild Alzheimer’s disease (AD). For example, Heyn [21] demonstrated an improvement in overall emotional mood in a small sample of 13 individuals with mild AD, using an intervention that included imagery with a warm-up session of seated exercise. Cognitive scores, as measured by the Menorah Park Engagement Scale, remained stable over the 6 months following the intervention. In our study, we assessed cognitive ability using the Walking While Talking Test, a dual-task measure commonly used with elderly individuals with AD [16]. Hussey et al. [22] noted that individuals with mild AD can perform basic visual imagery, but struggle with complex tasks related to verbal recognition.

In AD, visual imagery is considered a central component of the memory system, playing a key role in the experience of memory reliving [23]. Autobiographical memories often come to mind in the form of visual images, and these images are considered the primary format of the subjective experience of autobiographical memories [24]. Autobiographical memory is a system consisting of episodes recollected from an individual’s life, combining both episodic memory and semantic memory. As such, it is a type of explicit memory. Although explicit memory deteriorates in individuals with dementia, implicit memory—which involves learning without conscious awareness—tends to remain relatively preserved. This is particularly true for implicit memory associated with sensory or motor learning, as opposed to conceptual learning. According to Conway and Pleydell-Pearce [25], the creation of visual mental images aids autobiographical recall by facilitating quicker and easier navigation through the hierarchical structure of autobiographical memory. The role of imagery in autobiographical memory is further supported by studies showing that participants with high visual imagery abilities exhibit better autobiographical recall than those with low visual imagery abilities [26].

Research has examined the effectiveness of CAM techniques on cognitive status, emotional well-being, and quality of life in neurodegenerative diseases. A systematic review reported that action observation therapy and MI significantly improved quality of life in Parkinson’s disease patients after the treatment period in both the experimental and control groups, although these improvements were only maintained at follow-up in the experimental group [27]. Additionally, a study combining MI and action observation therapy was conducted with 30 patients with vascular cognitive impairment over 8 weeks. Cognitive function was assessed using the Montreal Cognitive Assessment (MoCA) and the Rivermead Behavioral Memory Test, which are simple clinical tools for detecting mild cognitive impairment and assessing memory skills. These tests were administered at baseline, 4 weeks, 8 weeks post-treatment, and 1 month after follow-up. In contrast, the present study used the Walking While Talking Test, a demanding dual-task measure of attention, to examine cognitive–motor interactions in elderly individuals. While memory issues are present in vascular cognitive impairment, they tend to be less pronounced than in Alzheimer’s-type dementia, which is the focus of our clinical population. The results of the study on vascular cognitive impairment suggested that combining cognitive training with MI and action observation therapy is an effective treatment for cognitive function [28]. Additionally, promising findings suggest that MI improves fatigue, mood, and quality of life in individuals with multiple sclerosis [29,30]. However, there is still limited understanding of how MI primes actual movements in multiple sclerosis patients [31].

Several studies have explored the psychological effects of MI on caregivers of individuals with dementia. For instance, one investigation revealed that 11 out of 12 caregivers assigned to the MI Therapy (MIT) group completed the program, attending nearly all weekly sessions. The waitlist group also completed the post-intervention assessments. Caregivers in the MIT group exhibited notably greater reductions in self-reported depression and anxiety symptoms after 4 weeks, in contrast to those in the waitlist group. Neuroimaging analysis revealed enhanced connectivity in the dorsolateral prefrontal cortex with a proposed emotion regulation network in the MIT group, whereas no such changes were detected in the waitlist group [32]. Caregivers showed improvements in well-being, mood, anxiety, and sleep, and many noted that MI helped them form and maintain healthier relationships. Some participants even described benefits in how they reacted to their social environment and how they perceived themselves more objectively from others’ perspectives. Specific elements of MI, such as self-compassion, self-care, and the ability to reflect on emotionally arousing challenges, may have mediated these improvements. Overall, family dementia caregivers reported positive effects of MI across multiple domains of well-being. Their reports showed that MI helps improving well-being, reducing non-mentalizing patterns of thought, and fostering balanced mentalization in their relationships with others [33].

Over 75% of individuals with dementia will exhibit behavioral and psychological symptoms (BPSDs) [34]. These symptoms can heighten the burden on caregivers and frequently result in earlier placement in nursing homes. A recent systematic review provided recommendations on assessing and managing BPSDs [35]. Leung et al. [36] reported the prevalence rates of depression, anxiety, and apathy in mild, moderate, and severe dementia as 38%, 41%, and 37%, respectively, for mild dementia; 38%, 41%, and 37% for moderate dementia; and 54%, 59%, and 43% for severe dementia. The prevalence of these symptoms did not vary by dementia stage or type. Among psychological symptoms, depression is one of the most frequently examined in studies of reflective exercise interventions for people with dementia. For instance, one study found a significant reduction in depression following 12 weeks of Silver Yoga compared to usual activity in people with dementia [37]. Symptoms of depression showed significant improvement after participating in a 12-week Kundalini yoga program [38]. Another study found that after three months, playing Mahjong led to a significant improvement in depression levels, surpassing the effects of tai chi and a handcraft intervention in older adults with moderate depressive symptoms residing in long-term care facilities [39]. However, these improvements were not sustained, as depression scores returned to baseline after 6 months. Other studies found no change in depression scores in individuals with dementia living in community settings or long-term care, despite baseline depression scores being within the normal range [40]. While the present study did not incorporate a follow-up evaluation for depression or anxiety, it similarly identified reductions in these symptoms after the application of MI. Notably, the Geriatric Depression Scale, a widely utilized tool in the referenced studies, was also employed in the present research to measure these outcomes.

Dementia-related anxiety has primarily been studied among cognitively healthy, community-dwelling adults [41]. However, one third of residents in elder care homes have anxiety [42], affecting both their overall quality of life and their care management. Few studies have utilized tools such as the Neuropsychiatric Inventory Questionnaire, the Generalized Anxiety Disorder Scale, or the Rating Anxiety in Dementia to evaluate anxiety in this clinical population [43,44]. In our study, we used the validated SAS test due to the lack of cross-culturally validated Greek scales specifically for anxiety in dementia. Future studies should consider using more specialized anxiety scales tailored for dementia.

Self-esteem significantly improved in people with dementia following 40 weeks of a multicomponent Tai Ji Quan intervention, in comparison to a sedentary control group [45]. Additionally, apathy, motivation, and passivity have been examined using the Apathy Evaluation Scale (AES) in people with dementia [46]. However, the present study focused only on depression and anxiety. Mood disturbances such as agitation, aggression, eating disturbances, and nighttime behavior were not investigated in this study. Therefore, future research should explore the effects of these mood disturbances on individuals within this clinical population.

Impairment of attention is a common early symptom of dementia, contributing to its progression, challenges in performing activities of daily living (ADLs), and disruptions in communication. Despite its significance, research on attention impairment in dementia remains limited [47]. This decline in attention may be linked to impaired balance, increasing the risk of falls. Poor balance is often associated with cognitive impairment, which results from cerebral cortex damage that affects motor functions. Hussey et al. [22] suggested that imagery might help individuals with mild AD overcome attention deficits and provide strategies for improving memory function [48]. Indeed, MI may enhance attention focus, leading to improved balance and a reduced risk of falls. However, the present study did not assess attention ability, highlighting the need for future research to address this critical aspect.

QOL is an essential factor for individuals with dementia. The effect of dementia on QOL can vary and it depends on the severity of the disease, the care received, and the individual’s personality prior to developing the condition. Mood is a key determinant of QOL at all stages of dementia severity [49]. Mood encompasses psychological well-being, depression, anxiety, happiness, self-esteem, and overall life satisfaction—all of which are crucial factors influencing QOL [50]. Lower QOL is strongly associated with depression and anxiety in individuals with dementia [51], while higher QOL is often observed in those with few depressive symptoms, irritability, and apathy [52]. Dementia patients faced challenges with mobility and social support, which can negatively affect their QOL, while better QOL is more closely linked to social interactions [53], improved general health [54], and reduced caregiver burden [55]. In the present study, MI was shown to enhance QOL, as assessed using the EuroQoL scale, acknowledging the absence of dementia-specific QOL instruments validated for the Greek patients.

Research highlights the positive effects of exercise on individuals with dementia. One example is the Preventing Loss of Independence through Exercise (PLIÉ) program, an integrative movement initiative designed for people with mild to moderate dementia attending adult day care programs [56]. PLIÉ emphasizes abilities that remain relatively intact in individuals with dementia, such as learning new movement sequences through repetition, utilizing procedural memory, perceiving bodily sensations in the present moment, and experiencing joy while forming meaningful social connections [57]. The program addresses five key areas that enhance the quality of life for individuals with dementia: physical function, cognitive function, well-being, social connection, and self-esteem [58].

Akram et al. [59] applied PLIÉ with veterans who had dementia, along with their family members. Participants reported improvements in four key domains: (1) physical (mobility, strength, energy), (2) psychological, (3) social, and (4) cognitive. These improvements were reflected in surveys from residents, staff, and family members, as well as clinical progress notes, with participants frequently noting gains in multiple areas. Similarly, Barnes et al. [60] implemented PLIÉ with women with mild to moderate dementia and their caregivers, reporting improvements in cognitive function and quality of life for all participants. Another study found significant reductions in behavioral and psychological symptoms of dementia (BPSD), as well as decreased caregiver distress and burden, following an 18-week PLIÉ intervention [59].

No studies until now have examined the effects of MI intervention on cognitive, emotional status, and quality of life in individuals with dementia. However, research has explored the benefits of participating in the OEP for people with dementia. The findings of the present study open new avenues for treating dementia, and future research should explore the potential benefits of OEP in this population. A significant strength of the current study was the large sample size, which allowed us to use valid assessments to evaluate quality of life, cognitive, and emotional status subjectively.

However, a limitation in this study may be the diverse age and sex of participants which have influenced the results, as it is challenging to recruit individuals with identical demographic characteristics. Moreover, this study targeted individuals at a specific stage of dementia, limiting the ability to generalize the findings to other stages of the disease. Another limitation is the dropout rate among participants, a common issue in older populations, which could have influenced the overall outcomes.

Future studies should clarify how changes in the central nervous system affect cognitive and emotional status in dementia and to identify the specific neurophysiological mechanisms responsible for these improvements. Additionally, studies should investigate whether there are differences in the effectiveness of visual versus kinesthetic imagery for individuals with dementia. It is necessary to explore the central nervous system changes that underlie cognitive and emotional improvements in dementia, as well as to compare the effects of visual versus kinesthetic imagery. It would also be valuable to investigate the impact of MI on other neurodegenerative diseases, to assess its effects on quality of life and emotional well-being and if these changes sustained for some months after the trial ended. Moreover, the development of cross-culturally validated tools in Greek, particularly for anxiety, apathy, and quality of life, would improve the accuracy of assessments in this population.

## 5. Conclusions

This study demonstrated that MI in individuals with early-stage dementia resulted in notable enhancements in cognitive function, emotional status, and quality of life compared to the control groups. It is essential to replicate these findings in subsequent research. Further investigations should explore the relationship between MI and conditions such as mild cognitive impairment, dementia, and AD. Additionally, exploring the psychophysiological mechanisms underlying MI’s therapeutic effects would be valuable. Future studies should also focus on identifying the neurophysiological processes that contribute to these improvements and investigate the varying effects of visual versus kinesthetic imagery on cognitive and emotional outcomes in dementia and other neurodegenerative disorders.

## Figures and Tables

**Table 1:** Characteristics of the participants.

Variables	Experimental Group (n = 55)	1st Control Group (n = 52)	2nd Control Group (n = 53)	One Way ANOVA F/X^2^
Age, years M (SD)	79.23 (6.58)	78.46 (7.25)	76.07 (7.50)	F(2, 2.87), *p* = 0.06
MMSE	23.45 (2.00)	22.73 (2.04)	23.41 (1.59)	F(2, 2.42), *p* = 0.09
Education				χ^2^(2, N = 160) = 1.27, *p* = 0.28
University, n (%)	32 (58.2)	23 (44.2)	24 (45.3)	
High School, n (%)	14 (25.5)	16 (30.8)	18 (34)	
Family status				χ^2^(2, N = 160) = 0.19, *p* = 0.82
Married n (%)	35 (63.5)	33 (63.5)	33 (62.3)	
Widow n (%)	17 (30.9)	13 (25)	17 (32.1)	
Divorced	1 (1.8)	1 (1.8)	2 (3.8)	
Live together				χ^2^(2, N = 160) = 0.14, *p* = 0.86
Husband/Wife	34 (61.8)	35 (67.3)	33 (62.3)	
Child	11 (20)	8 (15.4)	6 (11.3)	
Take care	-	3 (5.8)	1	
Alone	10 (18.2)	6 (11.5)	10 (18.9)	
Have children				χ^2^(2, N = 160) = 2.87, *p* = 0.06
Yes	53 (96.4)	52 (100)	44 (83)	
No	2 (3.6)	-	9 (17)	
Number of children				F(2, 2.67), *p* = 0.07
1 child	14 (25.5)	13 (25)	23.9 (43.4)	
2 children	33 (60)	32 (61.5)	19 (35.8)	
Profession				χ^2^(2, N = 160) = 2.37, *p* = 0.09
Civil servant	10 (18.2)	5 (9.6)	6 (11.3)	
Teacher	7 (12.7)	7 (13.5)	9 (17)	
Housework	6 (10.9)	5 (9.6)	7 (13.2)	
Private servant	5 (9.1)	16 (30.8)	6 (11.3)	
Chief engineering in military navy	3 (5.5)	1 (1.9)	1 (1.9)	
Salesman	1(1.8)	-	-	
Falls				F(2, 0.99), *p* = 0.37
No falls	32 (58.2)	33 (65.5)	33 (62.3)	
1 fall	14 (25.5)	10 (19.2)	16 (30.2)	
2 falls	8 (14.5)	9 (17.3)	4 (7.5)	

**Table 2:** Descriptive statistics of the Walking While Talking mistakes/time, the EuroQol total/on the measures ‘day, the Depression, the Anxiety (mean, SD).

Variables	1st Measurement (M,SD)	2nd Measurement (M,SD)	3rd Measurement (M,SD)	X^2^	df, *p*-Value
Walking While Talking_mistakes_	5.65 ± 5.74	4.02 ± 4.44	4.42 ± 4.78	14.95	2, 0.00
Walking While Talking_time_	23.20 ± 5.44	21.84 ± 5.43	21.02 ± 6.16	13.36	2, 0.00
EuroQuol total	6.12 ± 1.65	6.06 ± 1.49	5.80 ± 1.30	11.87	2, 0.00
EuroQuol on the day’s measurement	80.79 ± 8.61	82.06 ± 8.70	85.09 ± 9.67	25.59	2, 0.00
Depression	2.08 ± 2.20	1.92 ± 1.91	2.13 ± 2.24	6.54	2, 0.04
Anxiety	13.86 ± 4.67	12.96 ± 3.90	12.56 ± 4.32	39.90	2, 0.00

## Data Availability

The datasets presented in this article are not readily available because the data are part of an ongoing study and further analysis is required before they can be shared. Requests to access the datasets should be directed to vsakellari@uniwa.gr.
